# 3-(4-Chloro­benzo­yl)-6-(4-chloro­phen­yl)-2,4-dimethyl­benzonitrile

**DOI:** 10.1107/S1600536812036409

**Published:** 2012-08-25

**Authors:** Xin Wang, Yi-Min Zhang, Xue-Fei Jia

**Affiliations:** a School of Chemistry and Environmental Science, Henan Normal University, Xinxiang, Henan 453007, People’s Republic of China; b Xinyang Agricultural College, Xinyang, Henan 464000, People’s Republic of China

## Abstract

In the title compound, C_22_H_15_Cl_2_NO, the terminal chloro­benzene rings are oriented at 44.51 (15) and 86.06 (17)° with respect to the central polysubstituted benzene ring, and make a dihedral angle of 49.48 (17)°with each other. In the crystal, mol­ecules are linked by weak C—H⋯O and C—H⋯N inter­actions.

## Related literature

For background to the title compound, see: Ma (2003 ▶, 2005 ▶, 2007 ▶); Hoffmann-Röder *et al.* (2004 ▶). For related structures, see: Zhang *et al.* (2011 ▶); Fun *et al.* (2012 ▶); Jagadeesan *et al.* (2011 ▶).
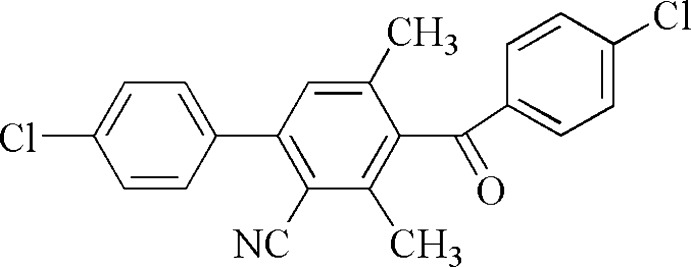


## Experimental

### 

#### Crystal data

C_22_H_15_Cl_2_NO
*M* *_r_* = 380.25
Monoclinic, 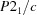

*a* = 7.8102 (12) Å
*b* = 30.032 (5) Å
*c* = 8.2054 (13) Å
β = 103.739 (2)°
*V* = 1869.6 (5) Å^3^
*Z* = 4
Mo *K*α radiation
μ = 0.36 mm^−1^
*T* = 296 K
0.39 × 0.33 × 0.23 mm

#### Data collection

Bruker SMART CCD area-detector diffractometer
Absorption correction: multi-scan (*SADABS*; Bruker, 2007 ▶) *T*_min_ = 0.873, *T*_max_ = 0.922
11684 measured reflections
3442 independent reflections
2013 reflections with *I* > 2σ(*I*)
*R*_int_ = 0.034

#### Refinement

*R*[*F* ^2^ > 2σ(*F* ^2^)] = 0.047
*wR*(*F* ^2^) = 0.121
*S* = 1.08
3442 reflections
237 parameters
H-atom parameters constrained
Δρ_max_ = 0.21 e Å^−3^
Δρ_min_ = −0.24 e Å^−3^

### 

Data collection: *SMART* (Bruker, 2007 ▶); cell refinement: *SAINT* (Bruker, 2007 ▶); data reduction: *SAINT*; program(s) used to solve structure: *SHELXTL* (Sheldrick, 2008 ▶); program(s) used to refine structure: *SHELXTL*; molecular graphics: *SHELXTL*; software used to prepare material for publication: *SHELXTL*.

## Supplementary Material

Crystal structure: contains datablock(s) I, global. DOI: 10.1107/S1600536812036409/xu5613sup1.cif

Structure factors: contains datablock(s) I. DOI: 10.1107/S1600536812036409/xu5613Isup2.hkl

Supplementary material file. DOI: 10.1107/S1600536812036409/xu5613Isup3.cdx

Supplementary material file. DOI: 10.1107/S1600536812036409/xu5613Isup4.cml

Additional supplementary materials:  crystallographic information; 3D view; checkCIF report

## Figures and Tables

**Table 1:** Hydrogen-bond geometry (Å, °)

*D*—H⋯*A*	*D*—H	H⋯*A*	*D*⋯*A*	*D*—H⋯*A*
C3—H3⋯N1^i^	0.93	2.61	3.305 (5)	132
C5—H5⋯O1^ii^	0.93	2.53	3.390 (5)	155

Symmetry codes: (i) 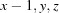; (ii) 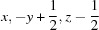.

**Fig. 1.**
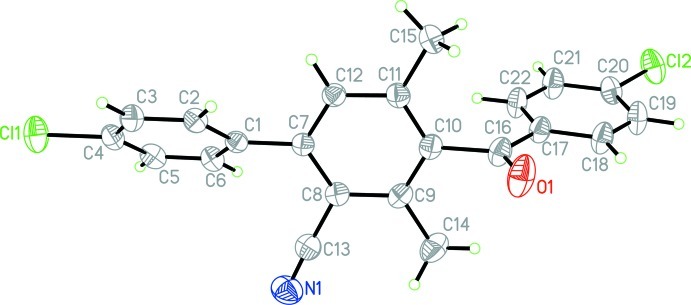
Molecular structure of the title compound, with displacement ellipsoids drawn at the 30% probability level.

**Fig. 2.**
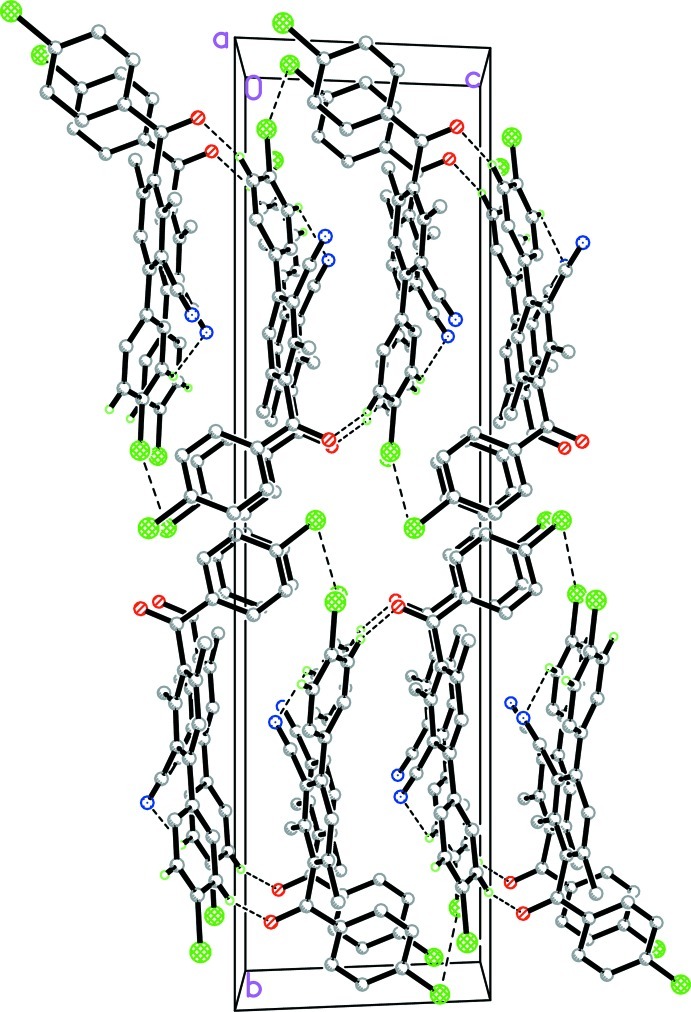
Crystal structure of the title compound, viewed along the a axis. Intermolecular C—H···O and C—H···N hydrogen bonds are shown as dashed lines, only H atoms involved in hydrogen bonds are shown.

## supplementary crystallographic information

### Comment

It has been well documented that benzenoid compounds are ubiquitous structural units in a wide variety of naturally occurring compounds and a plethora of pharmaceuticals. On the other hand, allene derivatives are powerful synthetic intermediates toward a plethora of important organic compounds and frequent building blocks of natural products (Ma, 2003, 2005, 2007; Hoffmann-Röder *et al.*, 2004). In this regard, Zhang *et al.* have developed a novel and efficient method for the preparation of polysubstituted benzenes by one-pot double Michael addition/*intramolecular* aldol reaction/ decarboxylation of 1,2-allenic ketones with cyanoacetate (Zhang *et al.*, 2011). Herein, we would like to report the structure of one of the products obtained by this method.

In the title compound (Fig. 1), all the bond lengths and bond angles are within normal ranges. The central polysubstituted benzene ring (C7—C12) forms dihedral angles of 44.51 (15)° and 86.06 (17)° with the other two chloro-substituted phenyl rings (C1—C6) and (C17—C22), respectively. And the dihedral angle between the two chloro-substituted phenyl rings, (C1—C7) and (C7—C12), is 49.48 (17)°. The mean plane of the ketone group is almost co-planar with the neighboring chloro-substituted phenyl ring (C17-C22).

In the crystal structure, the molecules are connected *via* C—H···O and C—H···N interactions.

### Experimental

A mixture of 1-(4-chlorophenyl)buta-2,3-dien-1-one (1 mmol), cyanoacetate (0.5 mmol) and K_2_CO_3_ (0.5 mmol) in acetone (5 ml) was refluxed for 15 min. Upon completion, the reaction mixture was cooled to room temperature, added with water (10 ml) and extracted with ethyl acetate. The combined organic phases were washed with brine, dried, filtered and concentrated under vacuum. The residue was purified by column chromatography on silica gel eluting with ethyl acetate/hexane (1:20 *v*/*v*) to give the title compound as Colorless solids with a yield of 80%. Single crystals, suitable for X-ray diffraction analysis, were obtained by slow evaporation of solvent from a petroleum ether-dichloromethane (2:1 *v*/*v*) solution.

### Refinement

The H atoms were included at calculated positions and were refined as riding atoms: C—H = 0.93 and 0.96 Å for aromatic and methyl H atoms, respectively, with *U*_iso_(H) =*x**U*_eq_(C), where *x* = 1.2 for aromatic H, and *x* = 1.5 for methyl H atoms.

### Crystal data

**Table:**

C_22_H_15_Cl_2_NO	*F*(000) = 784
*M**_r_* = 380.25	*D*_x_ = 1.351 Mg m^−^^3^
Monoclinic, *P*2_1_/*c*	Mo *K*α radiation, λ = 0.71073 Å
Hall symbol: -P 2ybc	Cell parameters from 3123 reflections
*a* = 7.8102 (12) Å	θ = 2.6–22.9°
*b* = 30.032 (5) Å	µ = 0.36 mm^−^^1^
*c* = 8.2054 (13) Å	*T* = 296 K
β = 103.739 (2)°	Block, colourless
*V* = 1869.6 (5) Å^3^	0.39 × 0.33 × 0.23 mm
*Z* = 4	

### Data collection

**Table:**

Bruker SMART CCD area-detector diffractometer	3442 independent reflections
Radiation source: fine-focus sealed tube	2013 reflections with *I* > 2σ(*I*)
Graphite monochromator	*R*_int_ = 0.034
phi and ω scans	θ_max_ = 25.5°, θ_min_ = 2.6°
Absorption correction: multi-scan (*SADABS*; Bruker, 2007)	*h* = −9→9
*T*_min_ = 0.873, *T*_max_ = 0.922	*k* = −36→36
11684 measured reflections	*l* = −9→9

### Refinement

**Table:**

Refinement on *F*^2^	Primary atom site location: structure-invariant direct methods
Least-squares matrix: full	Secondary atom site location: difference Fourier map
*R*[*F*^2^ > 2σ(*F*^2^)] = 0.047	Hydrogen site location: inferred from neighbouring sites
*wR*(*F*^2^) = 0.121	H-atom parameters constrained
*S* = 1.08	*w* = 1/[σ^2^(*F*_o_^2^) + (0.0235*P*)^2^ + 1.9216*P*] where *P* = (*F*_o_^2^ + 2*F*_c_^2^)/3
3442 reflections	(Δ/σ)_max_ < 0.001
237 parameters	Δρ_max_ = 0.21 e Å^−^^3^
0 restraints	Δρ_min_ = −0.24 e Å^−^^3^

### Special details

**Table:**

Geometry. All e.s.d.'s (except the e.s.d. in the dihedral angle between two l.s. planes)are estimated using the full covariance matrix. The cell e.s.d.'s are takeninto account individually in the estimation of e.s.d.'s in distances, anglesand torsion angles; correlations between e.s.d.'s in cell parameters are onlyused when they are defined by crystal symmetry. An approximate (isotropic)treatment of cell e.s.d.'s is used for estimating e.s.d.'s involving l.s. planes.
Refinement. Refinement of *F*^2^ against ALL reflections. The weighted *R*-factor *wR* and goodness of fit *S* are based on *F*^2^, conventional *R*-factors *R* are based on *F*, with *F* set to zero for negative *F*^2^. The threshold expression of *F*^2^ > σ(*F*^2^) is used only for calculating *R*-factors(gt) *etc*. and is not relevant to the choice of reflections for refinement. *R*-factors based on *F*^2^ are statistically about twice as large as those based on *F*, and *R*- factors based on ALL data will be even larger.

### Fractional atomic coordinates and isotropic or equivalent isotropic displacement parameters (Å^2^)

**Table:**

	*x*	*y*	*z*	*U*_iso_*/*U*_eq_	
C1	0.7995 (4)	0.21705 (10)	0.1502 (4)	0.0402 (7)	
C2	0.6497 (4)	0.20687 (10)	0.2063 (4)	0.0452 (8)	
H2	0.5945	0.2292	0.2535	0.054*	
C3	0.5808 (4)	0.16426 (11)	0.1934 (4)	0.0525 (9)	
H3	0.4806	0.1580	0.2318	0.063*	
C4	0.6621 (5)	0.13136 (10)	0.1233 (4)	0.0518 (9)	
C5	0.8084 (5)	0.14040 (11)	0.0632 (4)	0.0529 (9)	
H5	0.8606	0.1181	0.0131	0.063*	
C6	0.8767 (4)	0.18288 (10)	0.0779 (4)	0.0483 (8)	
H6	0.9767	0.1889	0.0386	0.058*	
C7	0.8714 (4)	0.26288 (10)	0.1663 (4)	0.0398 (7)	
C8	1.0517 (4)	0.27148 (10)	0.2239 (4)	0.0442 (8)	
C9	1.1199 (4)	0.31495 (11)	0.2343 (4)	0.0483 (8)	
C10	1.0020 (4)	0.35035 (10)	0.1930 (4)	0.0460 (8)	
C11	0.8210 (4)	0.34288 (10)	0.1389 (4)	0.0467 (8)	
C12	0.7596 (4)	0.29920 (10)	0.1238 (4)	0.0451 (8)	
H12	0.6394	0.2942	0.0838	0.054*	
C13	1.1720 (5)	0.23601 (12)	0.2910 (5)	0.0558 (9)	
C14	1.3152 (5)	0.32286 (14)	0.2910 (5)	0.0772 (12)	
H14A	1.3698	0.3164	0.2005	0.116*	
H14B	1.3639	0.3038	0.3845	0.116*	
H14C	1.3365	0.3534	0.3241	0.116*	
C15	0.6925 (5)	0.38100 (11)	0.0981 (5)	0.0646 (10)	
H15A	0.5752	0.3695	0.0592	0.097*	
H15B	0.7215	0.3991	0.0122	0.097*	
H15C	0.6988	0.3986	0.1969	0.097*	
C16	1.0722 (5)	0.39731 (11)	0.2256 (5)	0.0547 (9)	
C17	1.1277 (4)	0.42252 (11)	0.0920 (4)	0.0495 (8)	
C18	1.2013 (5)	0.46432 (11)	0.1290 (5)	0.0634 (10)	
H18	1.2167	0.4758	0.2367	0.076*	
C19	1.2517 (5)	0.48886 (12)	0.0070 (5)	0.0693 (11)	
H19	1.3029	0.5167	0.0326	0.083*	
C20	1.2263 (5)	0.47210 (11)	−0.1532 (5)	0.0589 (10)	
C21	1.1562 (5)	0.43075 (11)	−0.1909 (5)	0.0658 (11)	
H21	1.1412	0.4194	−0.2988	0.079*	
C22	1.1076 (5)	0.40580 (11)	−0.0673 (5)	0.0619 (10)	
H22	1.0608	0.3774	−0.0924	0.074*	
Cl1	0.57545 (16)	0.07788 (3)	0.10760 (15)	0.0838 (4)	
Cl2	1.28408 (16)	0.50406 (3)	−0.30697 (14)	0.0848 (4)	
N1	1.2698 (4)	0.20909 (12)	0.3525 (5)	0.0805 (11)	
O1	1.0819 (4)	0.41318 (9)	0.3638 (3)	0.0857 (9)	

### Atomic displacement parameters (Å^2^)

**Table:**

	*U*^11^	*U*^22^	*U*^33^	*U*^12^	*U*^13^	*U*^23^
C1	0.0442 (18)	0.0436 (17)	0.0329 (18)	0.0008 (14)	0.0092 (14)	0.0012 (14)
C2	0.049 (2)	0.0429 (18)	0.045 (2)	0.0041 (15)	0.0140 (16)	−0.0021 (15)
C3	0.055 (2)	0.051 (2)	0.054 (2)	−0.0044 (17)	0.0172 (17)	0.0015 (17)
C4	0.067 (2)	0.0420 (18)	0.045 (2)	−0.0002 (17)	0.0117 (18)	0.0021 (16)
C5	0.069 (2)	0.050 (2)	0.041 (2)	0.0109 (18)	0.0164 (18)	−0.0040 (16)
C6	0.057 (2)	0.0477 (19)	0.044 (2)	0.0038 (17)	0.0204 (17)	0.0003 (16)
C7	0.0453 (19)	0.0439 (18)	0.0323 (18)	−0.0010 (15)	0.0130 (15)	−0.0001 (14)
C8	0.045 (2)	0.0508 (19)	0.040 (2)	0.0009 (16)	0.0165 (15)	−0.0008 (15)
C9	0.046 (2)	0.061 (2)	0.039 (2)	−0.0086 (17)	0.0138 (16)	−0.0054 (16)
C10	0.057 (2)	0.0502 (19)	0.0314 (19)	−0.0099 (17)	0.0125 (16)	−0.0035 (14)
C11	0.058 (2)	0.0470 (19)	0.0354 (19)	−0.0021 (16)	0.0117 (16)	0.0022 (15)
C12	0.0446 (19)	0.0485 (19)	0.042 (2)	−0.0017 (16)	0.0095 (15)	0.0012 (15)
C13	0.050 (2)	0.062 (2)	0.059 (2)	0.0008 (19)	0.0207 (19)	0.0016 (19)
C14	0.054 (2)	0.085 (3)	0.090 (3)	−0.016 (2)	0.013 (2)	−0.010 (2)
C15	0.070 (3)	0.044 (2)	0.076 (3)	0.0014 (18)	0.011 (2)	0.0032 (18)
C16	0.063 (2)	0.055 (2)	0.048 (2)	−0.0154 (18)	0.0178 (18)	−0.0095 (18)
C17	0.059 (2)	0.0467 (18)	0.044 (2)	−0.0116 (17)	0.0144 (17)	−0.0070 (16)
C18	0.083 (3)	0.053 (2)	0.057 (2)	−0.023 (2)	0.021 (2)	−0.0158 (18)
C19	0.092 (3)	0.043 (2)	0.077 (3)	−0.022 (2)	0.030 (2)	−0.010 (2)
C20	0.070 (3)	0.045 (2)	0.066 (3)	−0.0041 (18)	0.026 (2)	0.0057 (18)
C21	0.094 (3)	0.053 (2)	0.056 (2)	−0.020 (2)	0.028 (2)	−0.0097 (18)
C22	0.082 (3)	0.049 (2)	0.058 (3)	−0.0240 (19)	0.022 (2)	−0.0079 (18)
Cl1	0.1082 (9)	0.0465 (5)	0.1010 (9)	−0.0146 (5)	0.0337 (7)	−0.0085 (5)
Cl2	0.1219 (10)	0.0556 (6)	0.0896 (8)	−0.0063 (6)	0.0505 (7)	0.0130 (5)
N1	0.065 (2)	0.083 (2)	0.095 (3)	0.018 (2)	0.023 (2)	0.014 (2)
O1	0.127 (3)	0.0814 (19)	0.0601 (19)	−0.0443 (17)	0.0442 (18)	−0.0276 (15)

### Geometric parameters (Å, º)

**Table:**

C1—C2	1.389 (4)	C12—H12	0.9300
C1—C6	1.393 (4)	C13—N1	1.144 (4)
C1—C7	1.481 (4)	C14—H14A	0.9600
C2—C3	1.382 (4)	C14—H14B	0.9600
C2—H2	0.9300	C14—H14C	0.9600
C3—C4	1.373 (4)	C15—H15A	0.9600
C3—H3	0.9300	C15—H15B	0.9600
C4—C5	1.374 (5)	C15—H15C	0.9600
C4—Cl1	1.736 (3)	C16—O1	1.216 (4)
C5—C6	1.377 (4)	C16—C17	1.479 (4)
C5—H5	0.9300	C17—C22	1.374 (4)
C6—H6	0.9300	C17—C18	1.384 (4)
C7—C12	1.389 (4)	C18—C19	1.373 (5)
C7—C8	1.398 (4)	C18—H18	0.9300
C8—C9	1.405 (4)	C19—C20	1.377 (5)
C8—C13	1.440 (5)	C19—H19	0.9300
C9—C10	1.395 (4)	C20—C21	1.363 (5)
C9—C14	1.504 (5)	C20—Cl2	1.729 (3)
C10—C11	1.396 (4)	C21—C22	1.384 (5)
C10—C16	1.514 (4)	C21—H21	0.9300
C11—C12	1.392 (4)	C22—H22	0.9300
C11—C15	1.507 (4)		
			
C2—C1—C6	117.6 (3)	C11—C12—H12	118.8
C2—C1—C7	120.4 (3)	N1—C13—C8	176.2 (4)
C6—C1—C7	122.0 (3)	C9—C14—H14A	109.5
C3—C2—C1	121.4 (3)	C9—C14—H14B	109.5
C3—C2—H2	119.3	H14A—C14—H14B	109.5
C1—C2—H2	119.3	C9—C14—H14C	109.5
C4—C3—C2	119.2 (3)	H14A—C14—H14C	109.5
C4—C3—H3	120.4	H14B—C14—H14C	109.5
C2—C3—H3	120.4	C11—C15—H15A	109.5
C3—C4—C5	121.0 (3)	C11—C15—H15B	109.5
C3—C4—Cl1	119.1 (3)	H15A—C15—H15B	109.5
C5—C4—Cl1	119.9 (3)	C11—C15—H15C	109.5
C4—C5—C6	119.3 (3)	H15A—C15—H15C	109.5
C4—C5—H5	120.3	H15B—C15—H15C	109.5
C6—C5—H5	120.3	O1—C16—C17	121.8 (3)
C5—C6—C1	121.5 (3)	O1—C16—C10	118.0 (3)
C5—C6—H6	119.3	C17—C16—C10	120.2 (3)
C1—C6—H6	119.3	C22—C17—C18	119.2 (3)
C12—C7—C8	117.5 (3)	C22—C17—C16	122.0 (3)
C12—C7—C1	120.3 (3)	C18—C17—C16	118.8 (3)
C8—C7—C1	122.2 (3)	C19—C18—C17	120.2 (3)
C7—C8—C9	121.9 (3)	C19—C18—H18	119.9
C7—C8—C13	120.4 (3)	C17—C18—H18	119.9
C9—C8—C13	117.4 (3)	C18—C19—C20	119.9 (3)
C10—C9—C8	118.4 (3)	C18—C19—H19	120.1
C10—C9—C14	121.1 (3)	C20—C19—H19	120.1
C8—C9—C14	120.5 (3)	C21—C20—C19	120.6 (3)
C9—C10—C11	121.0 (3)	C21—C20—Cl2	120.0 (3)
C9—C10—C16	118.5 (3)	C19—C20—Cl2	119.4 (3)
C11—C10—C16	120.2 (3)	C20—C21—C22	119.5 (3)
C12—C11—C10	118.8 (3)	C20—C21—H21	120.3
C12—C11—C15	119.9 (3)	C22—C21—H21	120.3
C10—C11—C15	121.3 (3)	C17—C22—C21	120.6 (3)
C7—C12—C11	122.3 (3)	C17—C22—H22	119.7
C7—C12—H12	118.8	C21—C22—H22	119.7
			
C6—C1—C2—C3	1.0 (5)	C16—C10—C11—C12	−174.6 (3)
C7—C1—C2—C3	−179.6 (3)	C9—C10—C11—C15	178.5 (3)
C1—C2—C3—C4	−0.2 (5)	C16—C10—C11—C15	5.1 (5)
C2—C3—C4—C5	−1.2 (5)	C8—C7—C12—C11	−0.9 (4)
C2—C3—C4—Cl1	179.9 (3)	C1—C7—C12—C11	179.2 (3)
C3—C4—C5—C6	1.8 (5)	C10—C11—C12—C7	2.4 (5)
Cl1—C4—C5—C6	−179.4 (3)	C15—C11—C12—C7	−177.3 (3)
C4—C5—C6—C1	−0.9 (5)	C7—C8—C13—N1	−119 (6)
C2—C1—C6—C5	−0.4 (5)	C9—C8—C13—N1	55 (6)
C7—C1—C6—C5	−179.9 (3)	C9—C10—C16—O1	−86.8 (4)
C2—C1—C7—C12	−44.8 (4)	C11—C10—C16—O1	86.8 (4)
C6—C1—C7—C12	134.6 (3)	C9—C10—C16—C17	92.8 (4)
C2—C1—C7—C8	135.3 (3)	C11—C10—C16—C17	−93.6 (4)
C6—C1—C7—C8	−45.3 (4)	O1—C16—C17—C22	−176.1 (4)
C12—C7—C8—C9	−1.9 (4)	C10—C16—C17—C22	4.3 (5)
C1—C7—C8—C9	178.1 (3)	O1—C16—C17—C18	3.5 (6)
C12—C7—C8—C13	171.8 (3)	C10—C16—C17—C18	−176.1 (3)
C1—C7—C8—C13	−8.3 (4)	C22—C17—C18—C19	0.7 (6)
C7—C8—C9—C10	3.0 (5)	C16—C17—C18—C19	−179.0 (4)
C13—C8—C9—C10	−170.8 (3)	C17—C18—C19—C20	1.0 (6)
C7—C8—C9—C14	−177.8 (3)	C18—C19—C20—C21	−1.9 (6)
C13—C8—C9—C14	8.4 (5)	C18—C19—C20—Cl2	178.0 (3)
C8—C9—C10—C11	−1.4 (4)	C19—C20—C21—C22	1.1 (6)
C14—C9—C10—C11	179.4 (3)	Cl2—C20—C21—C22	−178.9 (3)
C8—C9—C10—C16	172.1 (3)	C18—C17—C22—C21	−1.5 (6)
C14—C9—C10—C16	−7.1 (5)	C16—C17—C22—C21	178.1 (4)
C9—C10—C11—C12	−1.2 (5)	C20—C21—C22—C17	0.6 (6)

### Hydrogen-bond geometry (Å, º)

**Table:**

*D*—H···*A*	*D*—H	H···*A*	*D*···*A*	*D*—H···*A*
C3—H3···N1^i^	0.93	2.61	3.305 (5)	132
C5—H5···O1^ii^	0.93	2.53	3.390 (5)	155

Symmetry codes: (i) *x*−1, *y*, *z*; (ii) *x*, −*y*+1/2, *z*−1/2.
